# Adverse events in a large‐scale VMMC programme in Tanzania: findings from a case series analysis

**DOI:** 10.1002/jia2.25369

**Published:** 2019-07-31

**Authors:** Augustino Hellar, Marya Plotkin, Gissenge Lija, Amasha Mwanamsangu, Saidi Mkungume, Alice Christensen, Jeremiah Mushi, Michael Machaku, Thomas Maokola, Eric Mlanga, Kelly Curran

**Affiliations:** ^1^ Jhpiego Tanzania Dar es Salaam Tanzania; ^2^ Jhpiego Baltimore MD USA; ^3^ National AIDS Control Programme Ministry of Health, Community Development, Gender, the Elderly and Children Dar es Salaam Tanzania; ^4^ United States Agency for International Development Tanzania Dar es Salaam Tanzania; ^5^ Johns Hopkins University Bloomberg School of Public Health Baltimore MD USA

**Keywords:** voluntary medical male circumcision, adverse events, safety, Tanzania, quality, intervention, HIV prevention

## Abstract

**Introduction:**

Adverse events (AEs) rates in voluntary medical male circumcision (VMMC) are critical measures of service quality and safety. While these indicators are key, monitoring AEs in large‐scale VMMC programmes is not without challenges. This study presents findings on AEs that occurred in eight years of providing VMMC services in three regions of Tanzania, to provide discussion both on these events and the structural issues around maintaining safety and quality in scaled‐up VMMC services.

**Methods:**

We look at trends over time, demographic characteristics, model of VMMC and type and timing of AEs for 1307 males who experienced AEs among all males circumcised in Tabora, Njombe and Iringa regions from 2009 to 2017. We analysed deidentified client data from a VMMC programme database and performed multivariable logistic regression with district clustering to determine factors associated with intraoperative and postoperative AEs among VMMC clients.

**Results and discussion:**

Among 741,146 VMMC clients, 0.18% (1307/741,146) experienced a moderate or severe AE. The intraoperative AE rate was 2.02 per 100,000 clients, and postoperative rate was 2.29 per 1000 return clients. Multivariable logistic regression showed that older age (20 to 29 years) was significantly associated with intraoperative AEs (aOR: 3.51, 95% CI: 1.17 to 10.6). There was no statistical significant difference in AE rates by surgical method. Mobile VMMC service delivery was associated with the lowest risk of experiencing postoperative AEs (aOR:0.64, 95% CI: 0.42 to 0.98). AE rates peaked in the first one to three  years of the programme and then steadily declined.

**Conclusions:**

In a programme with robust AE monitoring methodologies, AE rates reported in these three regions were very low and declined over time. While these findings support the safety of VMMC services, challenges in reporting of AEs in a large‐scale VMMC programme are acknowledged. International and national standards of AE reporting in VMMC programmes are clear. As VMMC programmes transition to national ownership, challenges, strengths and learning from AE reporting systems are needed to support safety and quality of services.

## 1 Introduction

In March 2007, the World Health Organization (WHO) and the Joint United Nations Programme on HIV/AIDS (UNAIDS) recommended voluntary medical male circumcision (VMMC) for HIV prevention 1 based on evidence from three randomized clinical trials in South Africa, Kenya and Uganda 2, 3, 4. In 2009, the United States President's Emergency Plan for AIDS Relief (PEPFAR), led by the governments of these countries, began a massive scale‐up of VMMC services in 14 priority countries in sub‐Saharan Africa, including Tanzania 5. By 2017, 18.6 million males were circumcised in the 14 priority countries 5. These circumcisions will avert an estimated 1.1 million new HIV infections by 2030 5, but fell short of WHO's 2011 goal of 20.8 million circumcisions by 2016 5.

Tanzania's national HIV prevalence rate among adults was 5.0% in 2017 6, and an estimated 2.94 million circumcisions had been conducted by the same year 5. As a result of these efforts, Tanzania's male circumcision prevalence rate has increased, from 72% in 2010 7 to 80% in 2014 8. Like many other of the PEPFAR priority countries experiencing healthcare provider shortages 1, Tanzania has depended on “task shifting” (or role expansion) to non‐physician healthcare providers for provision of VMMC services 9 notably, allowing nurses to conduct circumcision.

In the decade following WHO's recommendation, as massive scale‐up of VMMC services occurred, quality and safety of have been closely monitored. WHO first released quality and safety guidance in 2008 and revised this guidance in 2018 10. Although male circumcision is a minor surgical procedure, risks and complications may be severe and occur rapidly, making both prevention and monitoring of adverse events (AEs) critical to VMMC programmes. Intraoperative AEs may be related to provider performance; postoperative AEs may be related to both provider and client factors, such as wound care and hygiene. Examples of intraoperative complications include excessive bleeding, pain, anaesthetic reactions, lacerations to the penile or scrotal skin, injury to the glans, excessive or insufficient skin removal, 10, 11 while postoperative AEs may include bleeding, hematoma, wound infection including severe infections such as tetanus or Fournier's gangrene, torsion of the penis, urethral fistulae, keloids, disfigurement and difficulty in micturition. Intraoperative AEs tend to be reported by documentation in a surgical register or immediately post‐operative, while postoperative AEs will usually be documented during a follow‐up visit occurring anywhere from discharge to one week following the procedure.

The closely monitored clinical trials in Uganda, Kenya and South Africa in 2005/06 reported rates of moderate and severe AEs as 3.6%, 1.5% and 3.8% respectively 2, 3, 4. AE rates from large‐scale programmes are known to have limited accuracy, due largely to reliance on the client to come back to report an AE, and documented poor adherence to follow‐up visit schedules 11, and healthcare provider reluctance to report AEs. Two separate studies in Kenya showed significantly higher AE rates in an active surveillance monitoring programme – while the rate of AEs in clinic monitoring systems was 2.1%, a rate of 7.5% was seen among clients in the active surveillance system 12.

Without contesting the potential limitation of underreporting, VMMC programmes have reported very low levels of AEs. For example, programmatic data from 44,868 VMMC clients in Zimbabwe showed an AE rate of 0.3% for surgical circumcision and 1.2% for device‐based (PrePex™) circumcision 13. AEs associated with surgical VMMC programmes tend to be comprise bleeding events in the zero to two day range, and shift to infections in day 3 onwards 11.

Our study reports on AEs associated with male circumcision in Tabora, Iringa and Njombe regions of Tanzania. We characterize AE type and severity among males circumcised through Tanzania's VMMC programme from 2009 to 2017 and assess the frequency and risk factors for AE by age, service delivery model, and surgical method. We discuss the challenges of programme monitoring of AEs and make recommendations on ways to improve monitoring, reporting of VMMC AEs. Our results are useful to policymakers, programme managers, and healthcare providers as VMMC programmes provide services at scale, and as countries are looking into transition to national ownership while maintaining safety of services.

## 2 Methods

### 2.1 Country programme setting

In Tanzania, approximately 80% of males aged 15 to 49 years are circumcised, with lower prevalence in some regions. In 2007/2008, male circumcision prevalence was 37.7% for Iringa and Njombe regions and 42.8% for Tabora 8.

With funding from PEPFAR through USAID, Tanzania's Ministry of Health, Community Development, Gender, the Elderly and Children (MOHCDGEC), launched VMMC services for HIV prevention in 2009 7. Jhpiego, an affiliate of Johns Hopkins University, implements the VMMC programme by providing technical support including training of MOHCDGEC VMMC providers, (who provide circumcision) equipment and supplies, demand creation and quality assurance. VMMC clients, aged 10 to 49 years, are offered HIV testing on an opt‐out basis. Following circumcision, the client is requested to return to where he was circumcised for two follow‐up visits – first within 48 hours and second within a week. VMMC services are offered at static sites in government health facilities, through mobile units that provide services in the community, and at campaign/outreach sites temporarily set up in health facilities or other community sites 7. After eight years of programme implementation, Iringa and Njombe regions have attained saturation (most males aged 15 to 29 are circumcised). These regions now have started Early Infant Male Circumcision (EIMC) services to help maintain this high coverage of male circumcision.

### 2.2 Study design and participants

We conducted a case series analysis of males in Iringa, Njombe and Tabora Regions in Tanzania who experienced circumcision‐related AEs, from September 2009 to December 2017.

### 2.3 Data collection

The Tanzania VMMC programme monitors AEs as recommended by WHO 14, classifying intraoperative and postoperative AEs by type and severity (mild, moderate or severe). When an AE occurs, a provider completes an MOHCDGEC AE form and stores it at the health facility where the circumcision was performed; if the facility is an outreach site, the provider files this form at the site's “parent” health facility. All client data are entered into a de‐dentified, client level database which is subject to rigorous quality control systems. This includes both surgery‐related and follow‐up visit information.

Intraoperative AEs occur during the circumcision procedure, as well as those which occur immediately post‐operatively. AEs which are reportable to the MOHCDGEC include severe pain, excessive bleeding, anaesthetic‐related event, excessive removal of skin, penile lacerations and glans amputation. Postoperative AEs are identified during the follow‐up visit(s), and those reportable to MOHCDGEC (at 48 hour visit) include pain, excessive bleeding, excessive or insufficient skin removal, swelling/hematoma, infection including Fournier's gangrene or tetanus, delayed wound healing, wound disruption, unsatisfactory appearance, and (at one week follow‐up visit or beyond) torsion of the penis, erectile dysfunction, undesirable sensory changes, keloids and psychobehavioral problems.

### 2.4 Data analysis

Descriptive statistics were calculated. Only moderate or severe AEs were included in the analysis. The intraoperative AE rate is the number of clients who experience AEs out of all clients circumcised, and the post‐operative AE rate is the number of clients who experience AE out of all circumcised clients who return for at least one follow‐up visit. We used logistic regression models with robust sandwich estimates to account for within‐district VMMC uptake variability to determine factors associated with AEs. All variables associated with AE rates in the univariate analysis (at the *p *<* *0.20 level) were included in the multivariable logistic regression models. Associations were estimated using odds ratios with 95% confidence intervals (CI). Finally, associations were examined at a significance level of *p *<* *0.05 (two‐sided test).

### 2.5 Ethics and consent

The analysis of these data received an “exempt” determination from Johns Hopkins Bloomberg School of Public Health Institutional Review Board (IRB#00004168) and was conducted with oversight from the National Institute of Medical Research (NIMR/HQ/R.8a/Vol.IX/2967). Written consent is needed to undergo male circumcision in Tanzania (adults) or signature of a parent or guardian (minors). No additional consent was obtained in relation to the secondary data analysis of deidentified programme data.

## 3 Results and discussion

In Iringa, Njombe and Tabora regions, 741,146 males were circumcised in the VMMC programme from September 2009 to December 2017. Over three‐quarters (78.3%) of the clients were between 10 and 19 years, and a majority (88.9%) were circumcised through campaign or outreach services (Table 1). The most frequently used circumcision method was forceps‐guided (59.7%), followed by dorsal slit (40.2%) (in 2014 use of forceps‐guided method was prohibited for VMMC clients aged below (15 years). Among all clients, 0.18% (1322/741,146) experienced a moderate or severe AE. Fifeteen intraoperative AEs were reported – an AE rate of 2.02 per 100,000 clients (15/741,146). A total of 1307 moderate and severe postoperative AEs were reported, half (46.9%) of which were severe. The AE ratio was 0.23% per 1000 clients who returned for at least one follow up visit (1454/571,964). The most common intraoperative AE was excessive bleeding (73.3%) – the most common postoperative AE was infection (77.9%).

**Table 1:** Background characteristics of VMMC clients, Tanzania 2006 to 2017 (N = 741,146)

Background characteristics	Frequency	Percent
Overall	741,146	100
Age group		
10 to 14	382,263	51.6
15 to 19	197,825	26.7
20 to 29	115,639	15.6
30+	45,268	6.1
Surgical method		
Dorsal slit	297,705	40.2
Forceps guided	442,200	59.7
PrePex	970	0.13
Sleeve resection	120	0.02
Service model		
Campaign/outreach	658,834	88.9
Routine/fixed	65,798	8.9
Mobile	16,363	2.2
Adverse events ratios		
Intra Op AE	15	0.002
Post Op AE^a^	1307	0.229
AE rates		
Intra‐operative AE	15	2.02/100,000
Post‐operative AE^a^	1307	2.29/1000
AE severity^b^		
Severe	621	46.9
Moderate	701	53.1
AEs by region^b^		
Iringa	542	41.0
Njombe	400	30.3
Tabora	380	28.7
AE occurrence timing^b^		
Intraoperative	15	1.1
<2 Days	56	4.2
2 to 7 Days	1075	81.3
7 + Days	176	13.3
AEs by calendar year^b^		
2009	0	0.0
2010	164	12.4
2011	116	8.8
2012	314	23.8
2013	187	14.2
2014	313	23.7
2015	152	11.5
2016	42	3.2
2017	34	2.3

^a^Calculated among 571,817 who returned at least for one follow‐up visit; ^b^includes both intra and post‐operative AEs.

**Figure 1:**
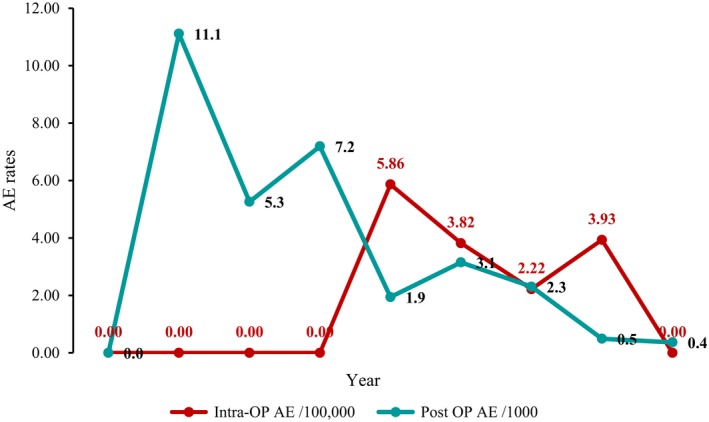
Intraoperative and postoperative AEs in Tabora, Iringa, and Njombe Regions, 2009 to 2017. AE, adverse event; OP, operative

The postoperative AE rate was the highest in 2010 at 11.1 per 1000 discharges, but has steadily declined over time; the intraoperative AE rate peaked in 2013 at 5.83 per 100,000 clients and dropped in the years following (Figure 1).

Excessive bleeding (n = 11) was the AE reported most often in males aged 20 to 29 years (Table 2). Glans injuries and excessive skin removal were infrequent, each occurring two times. Postoperative AEs occurred more commonly among younger males, and infections were the most common (n = 1019), accounting for half (50%) of AEs reported among boys aged 10 to 14 years. Bleeding was reported among 146 clients, with 39.7% occurring among boys aged 10 to 14 years. Swelling of the penis or scrotum was reported amongst 109 clients, with the highest (43.1%) incidence among males aged 15 to 19 years.

**Table 2:** Type of intraoperative and postoperative AE by age in Tabora, Iringa and Njombe Regions, 2009 to 2017

Type of AE	Age group (years)			
	10 to 14 n (%)	15 to 19 n (%)	20 to 29 n (%)	≥30 n (%)
Intraoperative AEs				
Bleeding problems	2/11 (18.2)	2/11 (18.2)	4/11 (36.3)	3/11 (27.3)
Damage to penis	1/2 (50.0)	0/2 (0.0)	1/2 (50.0)	0/2 (0.0)
Excess skin removal	0/2 (0.0)	1/2 (50.0)	1/2 (50.0)	0/2 (0.0)
Postoperative AEs				
Infections	519/1019 (50.9)	290/1019 (28.5)	142/1019 (13.9)	68/1019 (6.7)
Bleeding or blood soiling the bandage	58/146 (39.7)	41/146 (28.1)	36/146 (24.7)	11/146 (7.5)
Persistent pain	5/16 (31.2)	6/16 (37.5)	4/16 (25.0)	1/16 (6.3)
Swelling of penis or scrotum	21/109 (19.3)	47/109 (43.1)	35/109 (32.1)	6/109 (5.5)
Other	13/17 (64.7)	5/17 (23.5)	4/17 (11.7)	0/17 (0.0)

AE, adverse event.

Older age (being at least 20 years or older) was significantly associated with experiencing intraoperative AEs, and the risk increased with age (aOR:6.37, 95% CI: 1.57 to 25.8) (Table 3). Neither surgical method nor service delivery model was significantly associated with intraoperative AEs. The was no statistical significant difference in AE rates by surgical method. Mobile VMMC service delivery was associated with the lowest risk of experiencing postoperative AEs (aOR:0.64, 95% CI: 0.42 to 0.98).

**Table 3:** Factors associated with intraoperative and postoperative AEs in Tabora, Iringa and Njombe Regions, 2009 to 2017

Factors	Intraoperative AEs			Postoperative AEs		
	AEs/clients (% of all clients experiencing AE)	aOR [95% CI]	*p*‐value	AEs/clients (% of all clients experiencing AE)	aOR [95% CI]	*p*‐value
Age group (years)						
10 to 14	3/382,263 (0.001)	Reference		614/302,898 (0.2)	Reference	
15 to 19	3/197,825 (0.002)	1.87 [0.37 to 9.43]	0.448	388/145,228 (0.3)	1.02 [0.90 to 1.16]	0.710
20 to 29	6/115,639 (0.01)	6.37 [1.57 to 25.8]	0.010	219/87,899 (0.3)	0.90 [0.85 to 1.16]	0.935
≥30	3/45,268 (0.01)	8.44 [1.68 to 42.5]	0.009	86/35,792 (0.3)	0.89 [0.71 to 1.13]	0.348
Surgical method^a^						
Dorsal slit	1/10,708 (0.01)	Reference		29/8376 (0.4)	Reference	
Forceps‐guided	9/399,698 (0.002)	0.13 [0.02 to 1.22]	0.075	1054/291,214 (0.4)	0.98 [0.67 to .42]	0.901
PrePex^™^	0/968 (0.0)	‐		1/770 (0.1)	0.34 [0.05 to 2.58]	0.302
Sleeve resection	0/109 (0.0)	‐		0/97 (0.0)	‐	
Service delivery model						
Campaign/outreach	12/658,834 (0.002)	Reference		1168/513,887 (0.2)	Reference	
Routine/fixed	3/65,798 (0.005)	2.27 [0.64 to 8.09]	0.207	119/45,566 (0.3)	1.03 [0.85 to 1.25]	0.633
Mobile	0/16,363 (0.0)	‐		20/12,364 (0.2)	0.64 [0.42 to 0.98]	0.046

aOR, adjusted odds ratio; AE, adverse event; CI, confidence interval.

a This analysis was done for data from 2009 to 2014, when there was no age limitation in surgical method. After 2014, FG was allowed only for clients aged 15 and above and we did not include aORs for that period.

## 4 Discussion

Preventing AEs and making circumcision safer for clients are highest priorities for VMMC programmes 9, 10. In this VMMC programme, 0.18% of all VMMC clients had a reported moderate or severe AE, similar to the 0.3% rate reported in a VMMC programme in Zimbabwe 13. These rates of AEs are well below the rate in the landmark research studies conducted in 2007 2, 3, 4. Both in studies 3, 4 and programmatic settings, 13 the majority of AEs reported from VMMC service are moderate in severity. While this points in the direction of a very safe service, it is acknowledged that AEs in the programmatic setting are under‐reported, as further discussed below.

Rate of post‐operative AEs in the three regions steadily decreased over time, and intraoperative declined after 2013. These data were designed to monitor services, not to evaluate causality. However, we note the connection between scale up of the VMMC programme and declining rates of both postoperative and intraoperative AEs. The number of VMMCs performed in 2013 (136,740) was nearly three times the number performed in 2011 (48,682) (unpublished programme data). Trends we have seen in our programmatic data reinforce what is known internationally regarding safety of VMMC procedures: for example, AEs were higher for clients circumcised using forceps‐guided compared to dorsal slit method, reflecting WHO's call for safer services by promoting dorsal slit in 2014.

There is some precedent to the link between increased scale and quality: in a multicountry study in Eastern and Southern Africa, the scale‐ up of VMMC services was tied to improvements in quality 15. This is not universal, as another study in South Africa raised questions about the effect of scale on quality 16. We do feel that quality of service and AE reporting in this programme increased over time, but only with a vigilant focus on systems to prevent and report AEs.

Large VMMC programmes almost certainly underestimate AEs due to mobility of services, loss‐to‐follow‐up of clients, reluctance of healthcare providers to report AEs, provider knowledge of AE classification and other reporting challenges. In Kenya, the AE rate was over twice as high among males followed up at home compared to males followed at the clinic 17. While active surveillance of AEs would be beneficial to increasing the safety of services, VMMC programmes funded by PEPAR are bound to funding restrictions on unit cost per circumcision, and active surveillance would substantially increase costs. An investment into active surveillance thus might be at odds with ambitious targets set for what is meant to be the last push of VMMC services. The reality of VMMC service delivery, especially in mobile services, means that there are likely clients who either do not return with an AEs, or decide to seek services elsewhere and are lost to our monitoring systems. Under‐reporting of known AEs is also a concern affecting surgical programmes: in South Africa, 42% of surgeons stated hesitance to report an AE due to the perception of poor performance 18. Lack of knowledge about reporting and related tools, busy clinic schedules and fear of perceived incompetence 19, 20 have also been documented as reasons surgeons do not report AEs. Anecdotal evidence from Tanzania suggests that in particular mild AEs often go unreported.

Two trends may converge in VMMC programming in the next five years. First, if ambitious goals are met of increasing circumcision prevalence to 85% of the eligible male population, fewer adult circumcisions will need to be performed in the future. Second, the responsibility of VMMC service provision will shift to the governments of the host countries. As programs transition to host country leadership, low‐cost but effective models of monitoring AEs must be used, documented and evaluated. This could be active surveillance applied in limited geographies or situations. Promising approaches to be explored include QI approaches which reduce stigma around AE reporting – in Malawi, an audit‐based QI programme reduced AE under‐reporting by 48% 21.

Our study had limitations. The primary limitation is not unique to the programme described: large‐scale VMMC programmes are likely to underreport AEs for reasons discussed. We are confident that our programme provided maximum support to sites accurately and diligently report AEs, and our data systems are well supported and subject to quality control. It is possible that the AE rates presented here – while low in relation to the actual – represent one of the best characterizations of a highly functional programmatic AE reporting system. The programme also faces logistical challenges in AE reporting, such as how to capture post‐operative AEs in mobile or “satellite” sites. However, a decade of VMMC program implementation in Tanzania means experience matching these challenges with solutions, such as mobile follow up teams and working with local healthcare providers to recognize, manage, treat or refer AEs.

## 5 Conclusions

In eight years of VMMC programme implementation in Tabora, Iringa and Njombe regions of Tanzania, programme reports indicate that 1307 of the 741,146 VMMC clients experienced a moderate or severe AE. Our experience with provision of such large volume of VMMC services leads us to both acknowledge the challenges of monitoring AEs, but to also underscore the capacity of programmes to both monitor and analyse AEs to improve quality of services. Analysis of the demographics, such as age, timing and service delivery model has helped strengthen our service provision of VMMC services. New, low cost models of AE prevention and reporting are recommended as countries transition to local leadership of VMMC programmes.

## Competing interests

The authors declare no competing interests.

## Authors’ contributions

AH and MP drafted the manuscript. TM managed the data and ran reports, and AM analysed the data. GL, AC, SM and EM provided input on the discussion and programme background. All authors read and reviewed the drafts of the manuscript.
